# A laser-engraved wearable gait recognition sensor system for exoskeleton robots

**DOI:** 10.1038/s41378-024-00680-x

**Published:** 2024-04-08

**Authors:** Maowen Sun, Songya Cui, Zezheng Wang, Huayu Luo, Huayong Yang, Xiaoping Ouyang, Kaichen Xu

**Affiliations:** 1 State Key Laboratory of Fluid Power and Mechatronic Systems, School of Mechanical Engineering, Zhejiang University, Hangzhou, 310027 China; 2 School of Information and Electrical Engineering, Hangzhou City University, Hangzhou, 310015 China

**Keywords:** Electrical and electronic engineering, Sensors

## Abstract

As a reinforcement technology that improves load-bearing ability and prevents injuries, assisted exoskeleton robots have extensive applications in freight transport and health care. The perception of gait information by such robots is vital for their control. This information is the basis for motion planning in assistive and collaborative functions. Here, a wearable gait recognition sensor system for exoskeleton robots is presented. Pressure sensor arrays based on laser-induced graphene are developed with flexibility and reliability. Multiple sensor units are integrated into an insole to detect real-time pressure at key plantar positions. In addition, the circuit hardware and the algorithm are designed to reinforce the sensor system with the capability of gait recognition. The experimental results show that the accuracy of gait recognition by the proposed system is 99.85%, and the effectiveness of the system is further verified through testing on an exoskeleton robot.

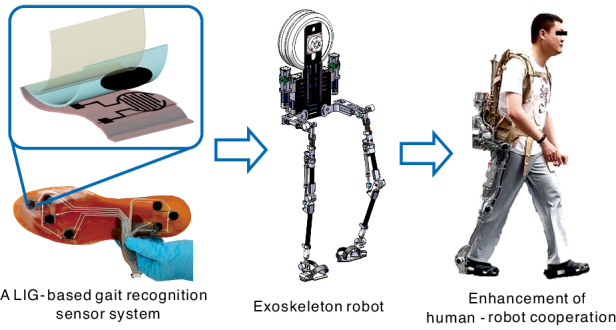

## Introduction

In recent years, exoskeleton robots have demonstrated wide applications in freight transport, disaster relief and rehabilitation due to their ability to enhance or extend human capabilities^1–3^. As a promising exoskeleton robot, the lower limb-assisted exoskeleton robot can follow the leg movement of a wearer and provide weight-bearing support. Unlike industrial robotic arms, the motion trajectory of a lower limb empowering exoskeleton robot cannot be generated by preprogramming or just based on the operating environment. Instead, it is planned according to the wearer’s movement intention and assistance requirements. In particular, gait information provides feedback on the walking status of a wearer, which affects the prediction of motion intention, assistance strategy and assistance effect. Therefore, gait recognition is one of the crucial tasks in human–robot cooperation involving lower limb exoskeleton robots^4,5^.

Wearable sensors that collect pressure, velocity and acceleration of the human foot are suitable for evaluating gait perception in exoskeleton robots. Generally, to construct such a sensor system, portability and compliance should be considered for seamless integration into the robot as well as for wearing comfort when moving limbs. However, typical sensor systems are currently based on rigid substrates of large sizes. In the past decade, the development of flexible electronics has provided sensors with a lightweight and favorable comfort^6–10^. Various flexible sensors, such as pressure sensors, temperature sensors and humidity sensors, have been designed and demonstrated to track human physical activities^11–19^. However, flexible sensors usually suffer from batch-to-batch variations as a result of limited fabrication techniques and methods, which hinders their application in real-life systems.

Customized laser direct writing technology is an efficient, high-precision, and green intelligent manufacturing technology that generates sensitive nanomaterials or functional micro/nanostructures via laser–matter interactions^20–25^. Using this approach, various flexible sensors, such as gas, strain, and pressure sensors and biosensors, have been demonstrated^26–33^. Laser-induced graphene (LIG) is one of the most useful materials for flexible sensors for sensing or interconnection electrodes and can be fabricated by one-step laser ablation of polyimide (PI) films^34–38^. By transferring LIG onto soft polymers, such as polydimethylsiloxane (PDMS), ecoflex, and hydrogels, composites can be generated for soft or flexible devices^39–42^.

In this work, a wearable LIG-based gait recognition sensor system is designed for application in exoskeleton robots to achieve real-time gait recognition. Through selective carbonization of PI films by laser processing, a customized intelligent insole composed of LIG/PDMS sensitive units and interdigital LIG electrodes was fabricated. Furthermore, the recognition accuracy of gaits under different conditions was improved using a machine learning algorithm. The practical application of the exoskeleton robot demonstrated that the LIG-based gait recognition sensor system can provide gait information with high accuracy for human–robot interaction control. An intelligent insole equipped with a highly sensitive pressure sensor array and algorithms has high potential for use in gait analysis and recognition in rehabilitation medicine.

## Results and discussion

**Fig. 1:**
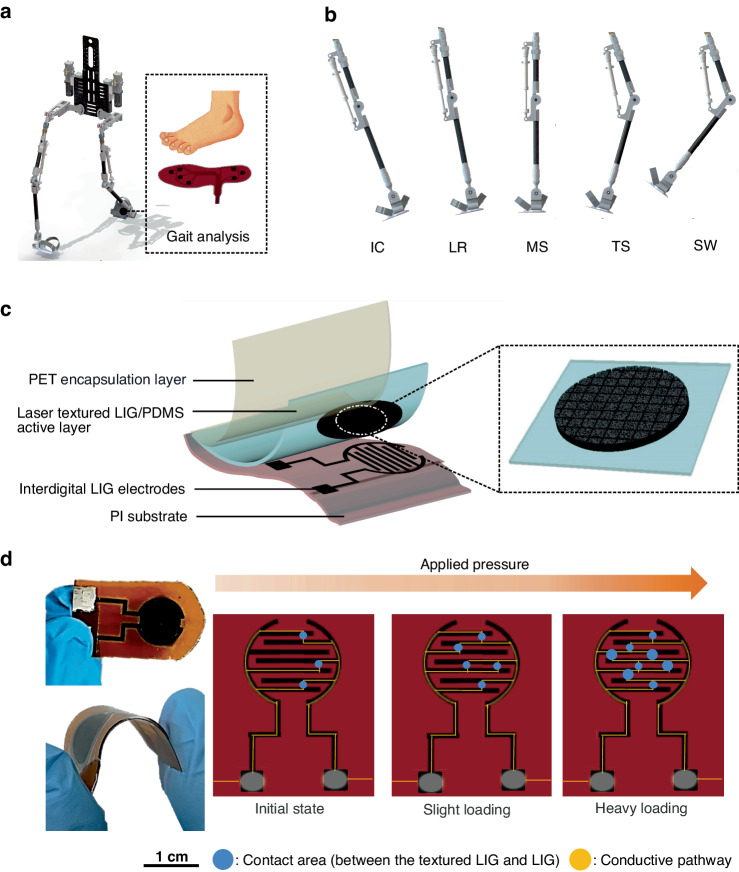
Applications and design of flexible pressure sensors for gait analysis. **a** Demonstration of an LIG-based pressure sensor sheet for gait analysis via integration with an exoskeleton robot. **b** Typical gait cycles include steps IC, LR, MS, TS, and SW. **c** Schematic of the pressure sensor composed of different layers, including LIG electrodes on a PI substrate, laser textured LIG/PDMS and PET encapsulation film. **d** Photos of the pressure sensor and a schematic diagram showing the working principle

During exoskeleton robot control, real-time gait information about the human body must be obtained. An intelligent insole system applied to detect the gait phase of an exoskeleton robot is shown in Fig. 1a. The system comprises seven units of pressure sensors located at foot stress points that reflect plantar pressure changes and pressure distribution states. In a single gait cycle, the movement of the feet can be categorized into several phases, including initial contact (IC), loading response (LR), mid-stance (MS), terminal stance (TS), and swing (SW), as shown in Fig. 1b. Initial contact between a heel and the ground is a transient process, revealing that the corresponding impact does not necessarily occur during each gait cycle. At the loading response step, the leg absorbs the impact until the forefoot lands on the ground. The foot is stationary during midstance and supports the body weight because the other foot begins to swing. When the heel lifts off the ground, the terminal stance starts and lasts, during which the toes remain touching the ground. Then, the foot will enter the swing phase. All the gait phases with touching points on the foot and ground are referred to as the stance phase. The sensor system was developed to accurately identify the aforementioned gait phases in real time.

The pressure sensors were constructed with three layers, comprising a PI film featuring LIG patterns, laser-textured LIG on a PDMS layer, and a PET encapsulation layer (Fig. 1c). Fig. S1 presents the fabrication procedures for the LIG-based pressure sensor. First, the LIG-sensitive layer and interdigital electrodes were created via laser ablation of PI films. Then, the sensitive LIG layer was transferred onto soft PDMS, followed by laser texturing to create additional hybrid structures. Finally, the LIG-based pressure sensor was obtained by aligning the LIG/PDMS sensing unit and interdigital LIG electrodes with the top PET encapsulation layer. The sensing mechanism of this LIG-based pressure sensor is similar to that of most pressure sensors (Fig. 1d). The variation in the contact area between the laser-textured LIG/PDMS electrode and the interdigital electrode leads to an electric current change.

**Fig. 2:**
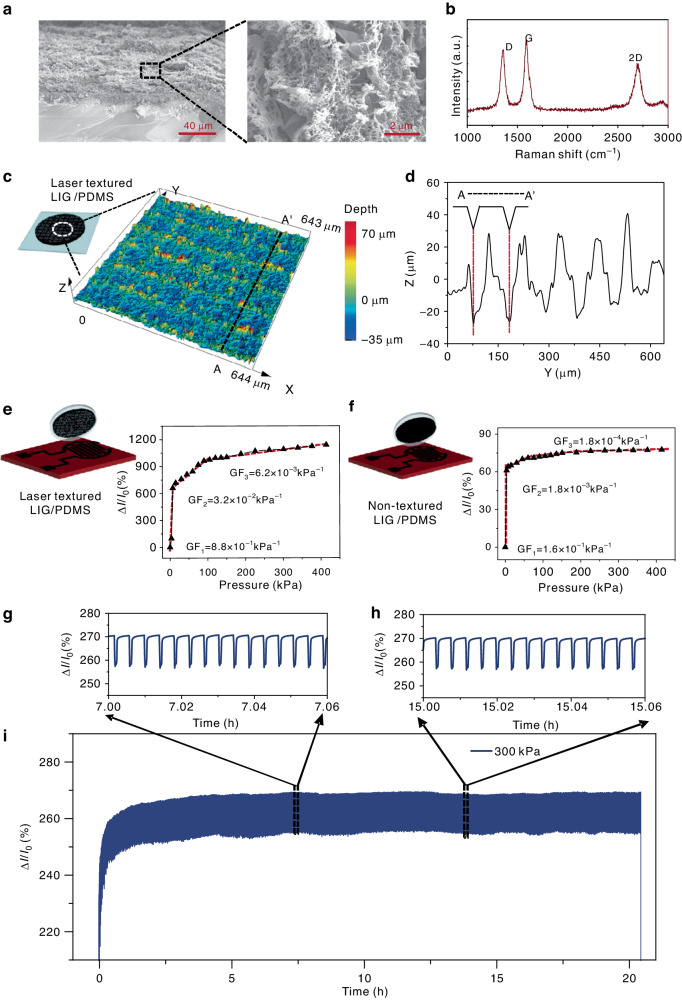
Characterization of flexible pressure sensors. **a** SEM image of the LIG. **b** Raman spectrum of the LIG. **c** The 3D surface morphology of LIG. **d** The corresponding depth of the microstructures. **e**, **f** Electric current changes in the sensors with and without LIG/PDMS laser texturing. **g**–**i** Long-term stability measurement (5000 cycles) under 300 kPa

To clearly depict the surface morphology of the LIG, scanning electron microscopy (SEM) images of the corresponding samples were obtained (Fig. 2a). The Raman spectra show the typical D (≈1350 cm^−1^), G (≈1580 cm^−1^), and 2D (≈2700 cm^−1^) peaks of LIG (Fig. 2b). The porous structure of LIG induced by pyrolysis gas during laser carbonization also contributes to the sensitivity of this pressure sensor^43^. To increase the sensitivity in response to pressure, the LIG/PDMS surface was textured by laser processing, resulting in hierarchical microstructures with a depth of ~40 μm (Fig. 2c, d).

To investigate the electric current variation in pressure sensors, it is essential to define the sensitivity. Here, sensitivity (*S*) is defined as

1 $$S=\frac{\triangle I/{I}_{0}}{P}$$

where *I*_*0*_ is the original electric current value of the sensor under no external force, *P* is the applied pressure, and *∆I* is the difference between the electrical current of the sensor under a certain pressure (*P*) and *I*_0_. In Eq. (1), S is proportional to ∆*I/I*_0_^44,45^.

To study the effect of micro/nanostructures on the performance of this sensor, the sensitivity of the laser-textured LIG/PDMS electrode (Fig. 2e) and untextured LIG/PDMS electrode (Fig. 2f) are evaluated. The pressure performance of laser-textured LIG/PDMS was shown to increase with increasing sensitivity, which could be ascribed to the increase in contact junctions between the laser-textured LIG/PDMS electrode and the interdigital LIG electrode. The laser engraving power of the microstructure on LIG/PDMS influences the initial electric current due to the penetration of laser irradiation. When the laser fluence is less than 8 J/cm^2^, the resistance does not change, indicating that the LIG patterns are still interconnected. Further increasing the laser fluence led to the rapid increase in the LIG resistance to 546.4% (Fig. S2). To optimize the pressure performance, the change in the electric current of this pressure sensor fabricated at different laser fluences was also characterized (Fig. S3). The pressure sensor fabricated at a laser fluence of 6.2 J/cm^2^ exhibited the highest sensitivity. This result occurs because the shallow structures of LIG/PDMS are patterned at a smaller laser fluence, resulting in a low difference in the contact area under pressure. In addition, at a larger laser fluence, the depth-to-width ratio of the laser-engraved pattern increases, but the effective contact area of the LIG is further reduced, leading to saturation of the electric current change at a low pressure.

In Fig. S4, the cycling stability of the flexible pressure sensor is reported under a constant pressure of approximately 100 kPa at frequencies of 0.05 Hz, 0.1 Hz, and 0.2 Hz after 10 cycles. In addition, cycling stability was also identified under various pressure values, including 50 kPa, 100 kPa, and 200 kPa at 0.1 Hz (Fig. S5). Notably, there is almost no clear degradation in pressure observed after ~5000 cycles of testing (Fig. 2g–i). The sensing performance of three replicates under a constant pressure of approximately 100 kPa at a frequency of 0.05 Hz is displayed in Fig. S6. The relationship between different pressure values (0–11 kPa) and the output voltage of the LIG/PDMS sensor unit was also tested (Fig. S7).

**Fig. 3:**
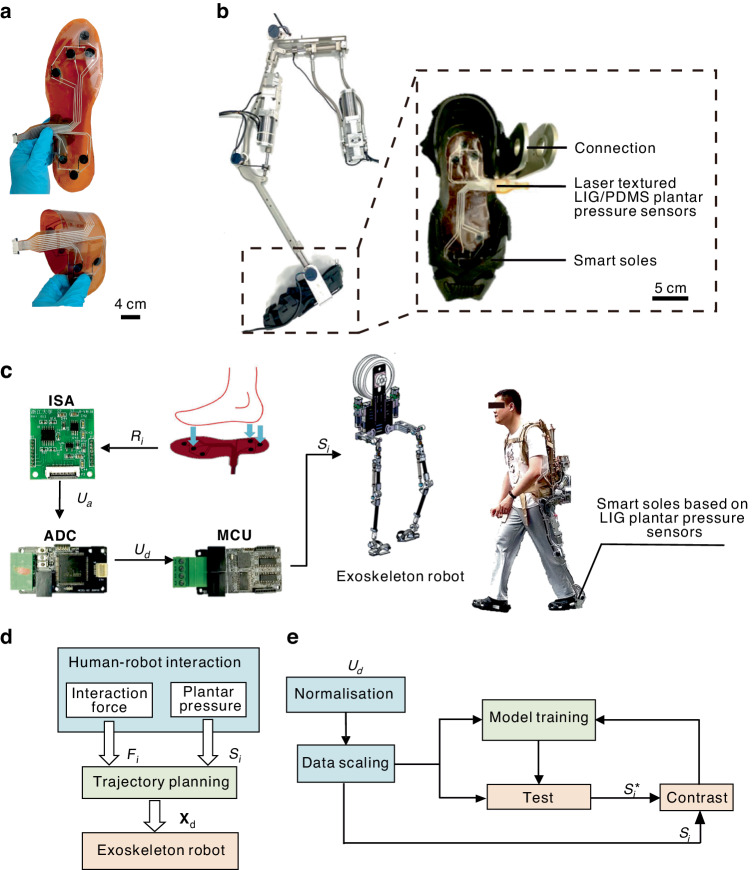
Design of the LIG-based gait recognition sensor system. **a** Photos of plantar pressure insoles. **b** Plantar pressure insoles integrated into the exoskeleton robot. **c** The hardware of the sensor system. **d** Control strategy of the exoskeleton robot. **e** Training process of the gait recognition model

After fundamentally characterizing a single LIG pressure sensor unit, multiple LIG pressure sensor units were assembled into an intelligent insole to measure plantar pressure, as shown in Fig. 3a. The intelligent insole is flexible and foldable to guarantee wearing comfort. The entire insole is 255 mm in length, 145 mm in width and 0.15 mm in thickness. The insole structure resembles that of a single pressure sensor. Three key plantar pressure position sampling points (CH1, CH2 and CH3, as shown in Fig. S8) were established according to the distribution characteristics of plantar pressure^46^. The end of the insole is equipped with 9-channel wiring terminals, which are connected to the circuit transceiver module and controller through ribbon cables (Fig. S9). The entire insole is embedded in the shoes of the exoskeleton robot, as shown in Fig. 3b.

The intelligent insole is embedded in the exoskeleton robot foot, which provides feedback on the plantar pressure state after wearing^3,47^. The insole connects with the shank rod of the exoskeleton robot through the connecting piece, as shown in Fig. 3b. Supporting hardware was developed for intelligent insoles to realize signal amplification, acquisition and processing. The hardware system is mainly composed of an input signal amplifier (ISA), an analog-to-digital converter (ADC) and a micro control unit (MCU). The signals of each module are transmitted through the controller area network (CAN) (Fig. 3c).

In contrast to industrial manipulators, quadruped robots and biped robots, the motion of exoskeleton robots does not depend entirely on the external environment or the robot’s planning. The control of the exoskeleton robot is a typical human-in-loop control mode. Figure 3d shows the control strategy of an exoskeleton robot when it is worn by an adult. The control process comprises three main components: the perception layer, the planning layer and the actuation control layer. The perception layer mainly perceives human–robot interaction information, including interaction force (*F*_*i*_) and gait perception information (*S*_*i*_). The planning layer generates the motion trajectory (*X*_*d*_*)* of the exoskeleton robot according to the perception information and ultimately executes the desired trajectory in the action control layer. The gait perception reflects the current motion state of the exoskeleton robot, and its accuracy determines the robot’s actuation control.

In Fig. 3e, the gait recognition model was obtained by the following steps. First, pattern calibration was performed on the plantar pressure information, and the collected plantar pressure data *U*_*d*_ were normalized. The data were then divided into a training set and a test set for offline model training and validation, respectively. The second step involved training the classification model. Based on the support vector machine (SVM) algorithm, a gait recognition model was generated by using the training set data. The model prediction results ${{S}_{i}}^{* }$ and the calibration results ${{S}_{i}}^{o}$ were compared, and parameter adjustments were subsequently performed to reduce judgment errors. Finally, the trained model was tested for its ability to simulate the actual gait of an exoskeleton robot in real time.

**Fig. 4:**
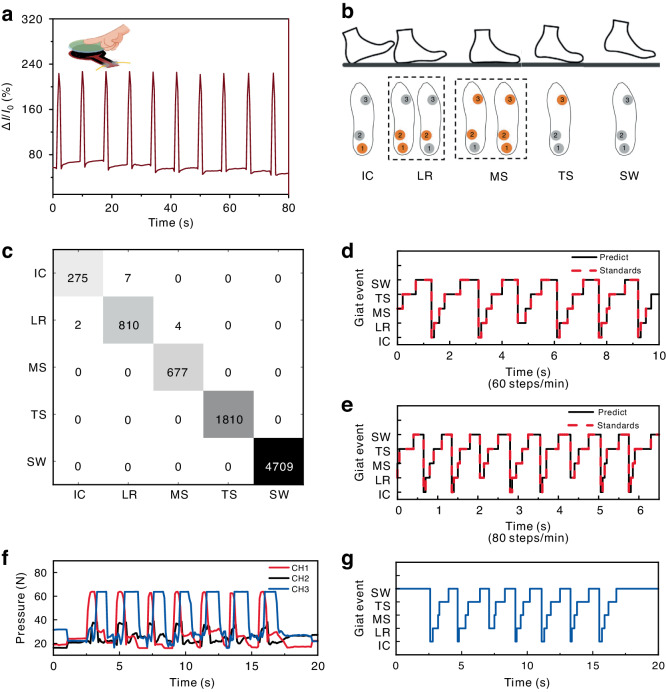
Applications of the gait recognition sensor system in an exoskeleton robot. **a** The relative electric current variation under pressure of the finger. **b** Relationships between gait phase and plantar pressure. **c**–**e** Comparison of the prediction and standard gait recognition test results. **f**, **g** Real-time application results of the exoskeleton robot integrated with the plantar pressure insole

To demonstrate the sensitivity of this pressure sensor, the changes in the electric current of this laser-textured LIG/PDMS sensor were tested under the pressure of a finger (Fig. 4a). It can be clearly seen that the Δ*I/I*_0_ value of this sensor decreases by ~175% under pressure. In addition, the mapping results of the pressure sensors located at three sampling points reflect the gait state. A mapping diagram between plantar pressure and gait, as shown in Fig. 4b, was established. The orange sampling points indicate that the area is under pressure. To evaluate the performance of the algorithm model, a gait recognition experiment was conducted on the existing collected data. Approximately 9160 sets of plantar pressure data in the calibration state were selected as the training set for model generation, and 7851 sets of data in the calibration state were selected as the validation group. The offline experimental results are shown in Fig. 4c. Only 13 groups of data were reported with errors in the prediction results and a high prediction accuracy of 99.85% was achieved.

Based on the offline experiments, a real-time walking gait judgment experiment was also carried out on a flat indoor surface. During this experiment, the volunteer wore intelligent insoles and walked at different gait frequencies (60 steps/min and 80 steps/min) to verify the effectiveness of the LIG-based gait recognition sensor system. The corresponding experimental results are shown in Fig. 4d, e. The results showed that the predicted results were consistent with the standard results even for different gait frequencies, which indicated the reliability of the LIG-based gait recognition sensor system. Moreover, the IC gait was not always captured during the walking process. Therefore, the LR gait phase is used as a sign of entering the standing phase, after which the exoskeleton robot works as an assisting agent.

Finally, the LIG-based gait recognition sensor system was applied in an exoskeleton robot on an asphalt road. The sensor load is transmitted to the ground through the hip and knee joints of the exoskeleton robot, so the insole mainly bears the wearer’s weight. A left leg gait cycle snapshot is shown in Fig. S10. Figure 4f shows the raw pressure data of three sampling points during the walking process. In each gait cycle, three plantar pressure units were sequentially activated following the order CH1, CH2 and CH3. The three-channel signals were stable and regular, which provided reliable data for the subsequent algorithm to evaluate. Figure 4g shows the gait results of a volunteer assisted by the smart exoskeleton robot during a real-time walking process.

## Conclusion

This work demonstrates a wearable sensor system with a timely feedback gait phase function to support an exoskeleton for human–robot interaction control. An embedded pressure sensor unit was successfully developed by a rapid and customized laser processing approach. The process realizes large-scale and repeatable fabrication of the conductive LIG patterns. Multiple sensor units and a printed circuit board are embedded into the insoles and integrated with the exoskeleton robot to reflect the gait of the wearer via pressure mapping. The SVM-based recognition algorithm is designed to realize highly accurate gait recognition. The experimental results show that the accuracy of the gait recognition sensor system based on the proposed system is 99.85%, and the reliability of the system is verified by practical applications of the exoskeleton robot. Future tasks include the integration of additional sensing modules, such as velocity and acceleration sensors, into the sensor system to comprehensively track the exoskeleton robot state. Overall, this LIG-based gait recognition sensor system is proposed to be applied to additional applications, such as rehabilitation or pathologic gait analyses.

## Experimental section

### Fabrication of laser-textured LIG/PDMS pressure sensors and intelligent insole

To create conducive LIG paths, the PI substrate was selectively carbonized by a CO_2_ laser (Universal Laser Systems, VLS3.50) under laser fluences varying from 4.3 to 6.9 J/cm^2^ with a wavelength of 10.6 μm. The scanning speed was 209 mm/s. In addition, the PET encapsulation layer was selectively cut in vector mode. To obtain the soft sensitive layer of the pressure sensor, the LIG was transferred to a PDMS substrate, and the microstructure was further textured by the laser. To fabricate the intelligent insole, silver electrodes were first screen printed on the PI film, which was cut into an insole-like shape by a laser. Then, the interdigital LIG electrode arrays were fabricated by laser carbonization, followed by the alignment of the laser-textured LIG/PDMS units. A PET film was finally applied for encapsulation.

### Fabrication of PCBs

Specialized input signal amplification circuits were designed for the intelligent insole. The function of ISA was mainly to convert the resistance (*R*_i_) of the pressure sensor unit of each channel into a voltage signal *U*_a_ and amplify it. In Fig. S6a, b, each ISA contains four amplification units, which convert the resistance value of each LIG pressure sensor unit into a voltage value through the principle of false short-false breaks. The relationship between the output voltage (*V*_out_) and the resistance of the LIG pressure sensor unit (*R*_LIG_) is *V*_out_ = 1.25 × (1 + *R*_LIG_/*R*_ref_). Figure S9a shows the schematic of ISA. The OPA4376 chip was chosen as the converter chip. OPA4376 was an operational amplifier chip containing four channels, and each channel was responsible for one-way voltage conversion. The function of the ADC module was to convert the analog signal *U*_a_ of ISA into the digital signal *U*_*d*_ that can be processed. Finally, the digital signals *U*_d_ of the three channels were transmitted to the MCU for calculation and processing.

### Characterization

The surface morphology of the laser-textured LIG surface was characterized by a thermal field emission scanning electron microscope (FE-SEM, Hitachi, SU-70 UHR) and a 3D measuring laser microscope (Olympus, OSL5000). A Raman spectroscope (Horiba, HR800) with a 532 nm wavelength was used to obtain the Raman spectra. Electric current measurements of the pressure sensors were performed using a digital multimeter (Keysight, 34470A). The pressure of the sensor was dynamically measured with a compression testing machine (Zhiqu Precision Instrument, ZQ-990B).

### Data acquisition and analysis

Five healthy adults ranging in age from 23 to 31 years were recruited as volunteers. The sampling frequency and control frequency bits were 100 Hz in the experiments. The size of the ISA was 20 mm × 24 mm. Each ISA module consisted of two amplification processing units. Four pressure sensor units were collected, and their signals were amplified. The basic principle of each ISA is shown in Fig. S9b. The ADC module mainly consisted of a power supply module and a voltage acquisition module. The power module and the voltage acquisition module are two important parts of the ADC module. The voltage acquisition module collects the voltage signal output from the sensor transmission module. The data were sent to the sensor network through the CAN bus. The size of the ADC module was 24 mm × 50 mm. The resolution was 16 bits, and signals from 8 channels were simultaneously collected. The available acquisition voltage ranged from −10 V to 10 V.

The gait recognition test data were collected through ISA and ADC, and the data analysis was conducted with MATLAB 2020b (Mathworks, MA, USA) software. The exoskeleton robot application test data were collected through ISA and ADC, and the data analysis was conducted in the MCU. The study protocol associated with wearable sensors was approved by the ethical committee of the College of Biomedical Engineering & Instrument Science, Zhejiang University ([2023]-66).

### Supplementary information

Supporting information
