# Supplementary material for: A laser-engraved wearable gait recognition sensor system for exoskeleton robots
Source: Microsyst Nanoeng. 2024 Apr 8;10:50. doi: 10.1038/s41378-024-00680-x (PMC11002036; doi:10.1038/s41378-024-00680-x)
Supplement: Supplementary file 1 — Supporting information [file 41378_2024_680_MOESM1_ESM.docx]

**A Laser-Engraved Wearable Gait Recognition Sensor System for Exoskeleton Robots**

**Supplemental Information**

^1^State Key Laboratory of Fluid Power and Mechatronic Systems, School of Mechanical Engineering, Zhejiang University, Hangzhou 310027, China

^2^School of Information and Electrical Engineering, Hangzhou City University, Hangzhou, 310015, China

^+^These authors contributed equally to this work

^*^Corresponding emails: [ouyangxp@zju.edu.cn](mailto:ouyangxp@zju.edu.cn), [xukc@zju.edu.cn](mailto:xukc@zju.edu.cn)


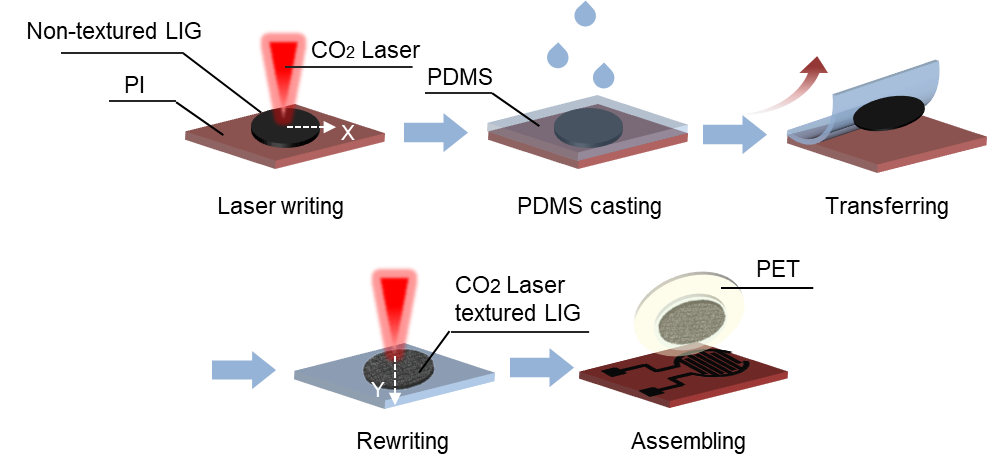


**Figure S1** Schematic of the fabrication procedures of the LIG-based pressure sensor.


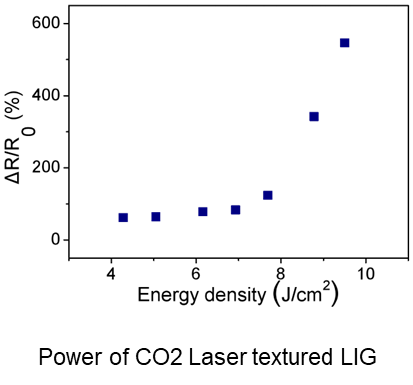


**Figure S2** Resistance change of the laser textured LIG/PDMS at different laser fluences.


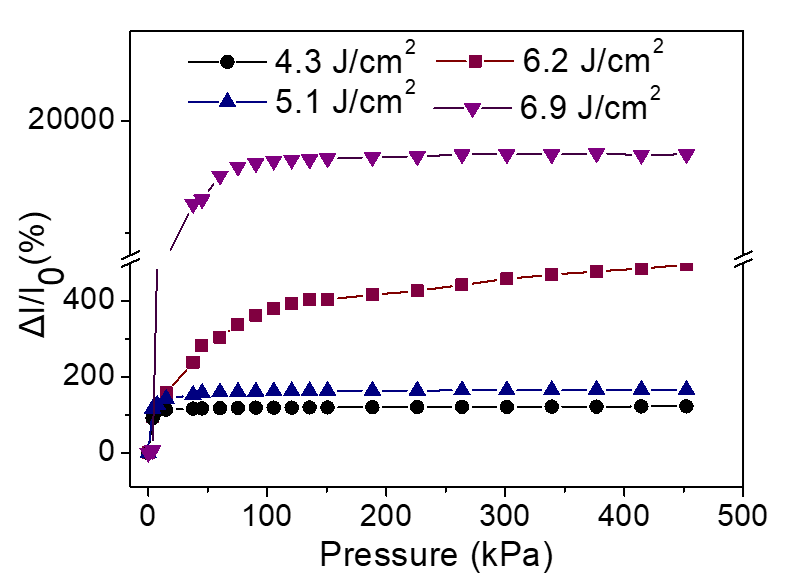


**Figure S3** Pressure sensing performance of the laser textured LIG/PDMS at different laser fluences.


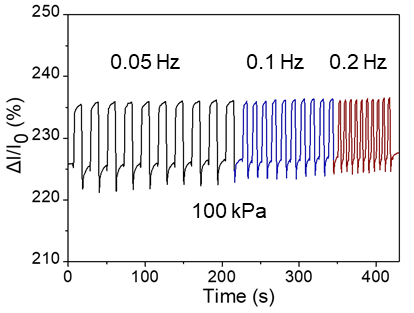


**Figure S4** Electric current at 100 kPa as a function of applied force frequency.


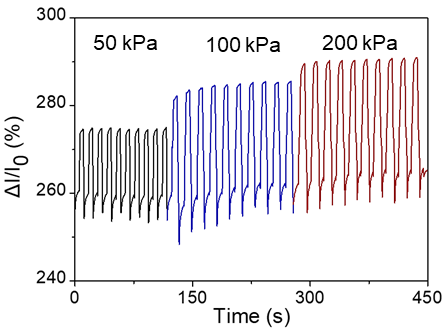


**Figure S5** Electric current change at 0.1 Hz as a function of pressure.

**
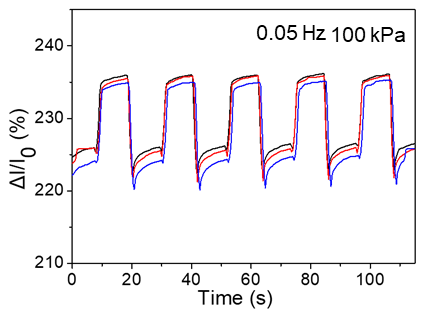
**

**Figure S6** Sensing performance of three replicates under a constant pressure of about 100kPa at the frequency of 0.05 Hz.


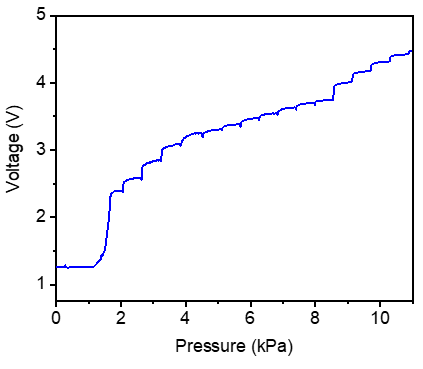


**Figure S7** The responses of the sensor to different pressures.


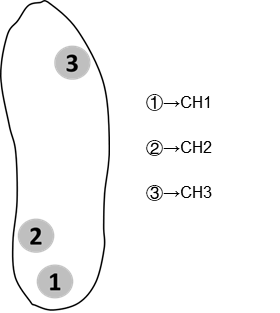


**Figure S8** The relationship between the LIG/PDMS sensor unit and signal channel.


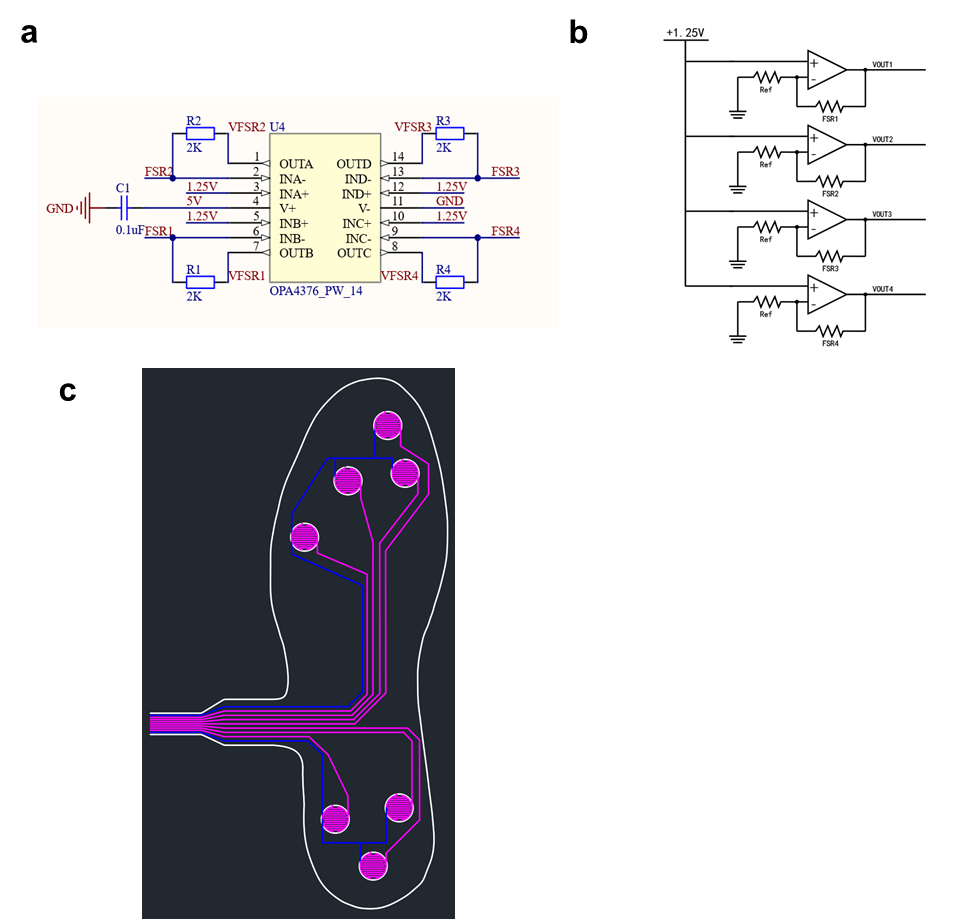


**Figure S9** The schematic of the circuit. **a** Schematic of the ISA. **b** Basic principle of ISA.

**c** Diagram of insole circuit.


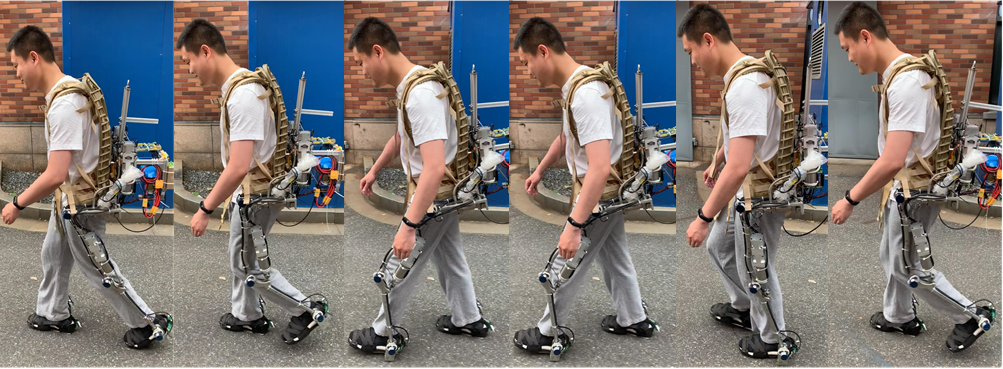


**Figure S10** Photo of a left leg gait cycle with the LIG-based gait recognition sensor system applied in the exoskeleton robot.
